# From the archives: Epidermal affairs—*EVER* links floral scent with cuticular waxes, while *SPL9* and *DEWAX* connect them to diurnal regulation, and *FIDDLEHEAD* takes on a function in the epidermis

**DOI:** 10.1093/plcell/koae231

**Published:** 2024-08-14

**Authors:** Christian Damian Lorenzo

**Affiliations:** Assistant Features Editor, The Plant Cell, American Society of Plant Biologists; Center for Plant Systems Biology, VIB, B-9052 Gent, Belgium; Department of Plant Biotechnology and Bioinformatics, Ghent University, B-9052 Gent, Belgium

## 2023: EVERlasting perfume: EVER links floral scent with cuticular wax

Like any living multicellular organism, plants have an outermost layer of cells known as the epidermis. The epidermis forms a boundary between plants and the external environment, and its cell walls are covered by a protective layer called the cuticle. This barrier, built from cutin polymers and waxes, confers several properties to plants, including water loss control, defense against biotic stresses, and gas transport facilitation (Arya et al. 2021). Research has hinted at a possible role of cuticle waxes with floral scent (Liao et al. 2021). Flower fragrances are the result of volatile compounds released into the environment through the cuticle. To gain a better understanding of floral scent mechanisms, Skaliter et al. (2023) studied the transcriptomic profiles of the adaxial epidermis of petunia (*Petunia × hybrida*) petals. As a result of their RNAseq analysis, the authors identified a gene they named *EPIDERMIS VOLATILE EMISSION REGULATOR* (*EVER*) to be highly expressed in the epidermis. Using a virus-based CRISPR/Cas9 system, the group generated petunia *EVER* knockout (KO) lines, which displayed high volatile emissions. Strikingly, RNA seq analysis of the *ever* KOs did not display major changes in known volatile biosynthesis genes but rather a severe enrichment in wax metabolism genes. Interestingly, though petunia *ever* KOs displayed altered epicuticular wax composition, no differences were observed in other cuticle properties such as its thickness or its water loss capacity. Therefore, *ever* KO leads to altered epicuticular wax profiles, affecting composition and, seemingly, the diffusion rates of different volatiles.

## 2019: From dusk till dawn: SPL9 and DEWAX govern diurnal regulation of wax synthesis

Epicuticular wax deposition is also a dynamic process that can be regulated by different external cues. Li et al. (2019) illustrated this point by showing how wax synthesis is photoperiodically regulated. Using yeast 1-hybrid screening (Y1H), the group identified *SQUAMOSA BINDING PROMOTER 9* (*SPL9*) as a potential regulator of *ECERIFERUM 1* (*CER1*) expression, a gene involved in cuticular wax component biosynthesis. *SPL9* is under the control of miR156 and is involved in multiple plant developmental and metabolic pathways (Xu et al. 2016). However, a direct link with wax biosynthesis has not been observed before. By examining the cuticular wax layer in *SPL9* and *miRNA156* mutants, suppressors, and overexpressing (OX) lines, the authors observed that *spl9* had a reduced wax load in the cuticle, while *SPL9OX* and lines with reduced miR156 activity showed the opposite effect. Thus, *SPL9* appears to be a positive regulator of wax biosynthesis. Further studies on the *SPL9* mode of action revealed that its upregulation in turn leads to increased expression of both *CER1* and *CER4*, thereby promoting wax biosynthesis. The authors also identified another gene involved in wax biosynthesis in their initial Y1H screen: *DEWAX*. While SPL9 binds to the promoter region of *DEWAX* to induce its expression, the DEWAX protein is an interactor and negative regulator of SPL9, thereby creating a negative feedback loop. Interestingly, both *SPL9* and *DEWAX* are diurnally regulated at the transcriptional and translational level, with longer day lengths corresponding with higher levels of wax accumulation. Therefore, this dynamic repression–activation transcriptional module established by SPL9 and DEWAX may play a role in allowing plants to optimize wax synthesis during day-night cycles.

## 1999: Rolling out FIDDLEHEAD function in the epidermis

**Figure 1.**
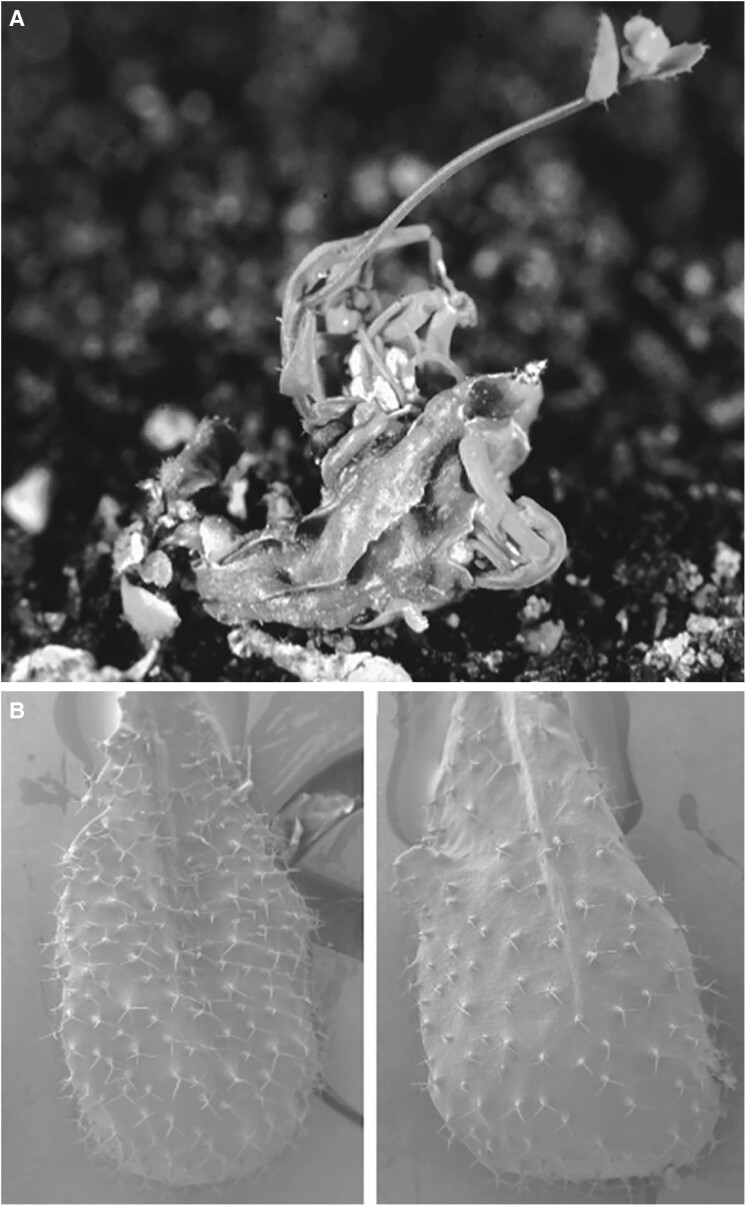
Phenotypes of the *fdh-3940* mutant. **A)** Fusion of leaves in plants homozygous for the *fdh-3940* allele. **B)** Effect of the *fdh* mutation on trichome differentiation: cryoscanning electron microscopy of Col-0 wild type (left) and *fdh-3940* leaf (right). Adapted from Yephremov et al. (1999), Figures 1 and 8.

Besides being the location for cuticular wax deposition, the epidermis itself plays an important role in plant development and organ shape. The epidermis is a single sheet of cells that derives from the L1 meristematic layer and serves essential functions, including protection, growth regulation, and facilitating environmental interactions (Zuch et al. 2022 ). L1 cells can re-differentiate into several epidermal types during ontogenesis to accomplish such a variety of roles. Yephremov et al. (1999) found roles for the *FIDDLEHEAD* (*FDH*) gene in cell adhesion and differentiation in the epidermis. The authors first identified an *Arabidopsis thaliana* transposon mutant with fusions between floral organs and leaves (Fig. 1). Benefitting from transposon tagging features, *FDH* was identified as the causative gene for the observed phenotypes. Through in situ hybridization, it was then observed that *FDH* expression was specifically enriched in L1 cells in meristems and inflorescences. Further characterization of stable *FDH* mutants revealed that these lines also carried fewer trichomes than the wild type (Fig. 1B). The protein product of *FDH* shows similarity to lipid biosynthesis enzymes. However, epicuticular waxes were not affected in the *fdh* mutant. The results obtained propose that *FDH* mutation affects the choice of cell fate in the epidermis resulting in cell fusions. Later research in Arabidopsis and *Antirrhinum majus* would reveal that *FDH*'s high expression may regulate cell separation and, conversely, its downregulation or suppression results in cell fusions in determinate reproductive structures (Efremova et al. 2004).

## Dive Curated Terms

The following phenotypic, genotypic, and functional terms are of significance to the work described in this paper:

SPL9 Gramene: At2g42200
SPL9 Araport: At2g42200

CER1 Gramene: AT1G02205
CER1 Araport: AT1G02205

FDH Gramene: AT5G14780
FDH Araport: AT5G14780

CER4 Gramene: AT4G33790
CER4 Araport: AT4G33790

flower AmiGo: PO:0009046

DEWAX Gramene: AT5G61590
DEWAX Araport: AT5G61590
